# Ribonuclease inhibitor 1 (*RNH1*) deficiency cause congenital cataracts and global developmental delay with infection-induced psychomotor regression and anemia

**DOI:** 10.1038/s41431-023-01327-7

**Published:** 2023-03-20

**Authors:** Carola Hedberg-Oldfors, Sanhita Mitra, Angela Molinaro, Kittichate Visuttijai, Linda Fogelstrand, Anders Oldfors, Fredrik H. Sterky, Niklas Darin

**Affiliations:** 1 Department of Laboratory Medicine, Institute of Biomedicine, Sahlgrenska Academy at the University of Gothenburg, Gothenburg, Sweden; 2 Wallenberg Centre for Molecular and Translational Medicine, University of Gothenburg, Gothenburg, Sweden; 3 Department of Clinical Chemistry, Sahlgrenska University Hospital, Gothenburg, Sweden; 4 Department of Pediatrics, University of Gothenburg, The Queen Silvia Children’s Hospital, Gothenburg, Sweden

**Keywords:** Paediatric neurological disorders, DNA sequencing

## Abstract

Ribonuclease inhibitor 1, also known as angiogenin inhibitor 1, encoded by *RNH1*, is a ubiquitously expressed leucine-rich repeat protein, which is highly conserved in mammalian species. Inactivation of *rnh1* in mice causes an embryonically lethal anemia, but the exact biological function of RNH1 in humans remains unknown and no human genetic disease has so far been associated with *RNH1*. Here, we describe a family with two out of seven siblings affected by a disease characterized by congenital cataract, global developmental delay, myopathy and psychomotor deterioration, seizures and periodic anemia associated with upper respiratory tract infections. A homozygous splice-site variant (c.615-2A > C) in *RNH1* segregated with the disease. Sequencing of RNA derived from patient fibroblasts and cDNA analysis of skeletal muscle mRNA showed aberrant splicing with skipping of exon 7. Western blot analysis revealed a total lack of the RNH1 protein. Functional analysis revealed that patient fibroblasts were more sensitive to RNase A exposure, and this phenotype was reversed by transduction with a lentivirus expressing *RNH1* to complement patient cells. Our results demonstrate that loss-of-function of RNH1 in humans is associated with a multiorgan developmental disease with recessive inheritance. It may be speculated that the infection-induced deterioration resulted from an increased susceptibility toward extracellular RNases and/or other inflammatory responses normally kept in place by the RNase inhibitor RNH1.

## Introduction

Ribonuclease inhibitor 1, also known as angiogenin inhibitor 1, encoded by *RNH1*, is a ubiquitously expressed Leucine-rich repeat (LRR) protein [1, 2]. RNH1 is a 50 kDa protein, that is widely expressed in mammalian cells and composed of 15 leucine-rich repeats that form a horseshoe-shaped overall structure [1, 3]. Despite being extensively characterized biochemically, its biological role remains unclear. A well-established biochemical function is to bind with high (femtomolar) affinity to ribonucleases, such as RNase1 (RNase A), RNase2, RNase4 and RNase5, thereby inhibiting their activity [1, 4, 5]. The cytotoxicity of different ribonucleases correlates inversely with their affinity toward RNH1, implying that only ribonucleases that evade RNH1 may be cytotoxic and fatal to the cell [1]. In addition to this protective function, RNH1 has been hypothesized to be involved in several different cellular pathways such as protein translation regulation by various mechanisms [5], in modulating inflammatory response by reducing the inflammasome activation [6] and as a scavenger for reactive oxidative species [7, 8]. Mice knockout for *Rnh1* die in utero during embryonic stage E8.5-E10 due to impaired development of the hematopoietic system and anemia [9]. The authors identified Rnh1 as a ribosome-associated protein that regulates translation of the erythropoietic transcription factor GATA1 [9]. To date, no disease-causing variants in humans have been reported in *RNH1*.

Here, we report a family with two siblings carrying a bi-allelic null variant in *RNH1* with a disease characterized by global developmental delay, muscle weakness and congenital cataracts. Psychomotor deterioration appeared to be induced by common infections during which seizures and transient macrocytic anemia were also observed. One of the siblings died of brain oedema during one such episode.

## Material and methods

### Genetic testing

For patient II:7, previous genetic analysis including chromosomal karyotyping and investigations for spinal muscular atrophy, Prader-Willi syndrome and myotonic dystrophy type 1 (DM1) were normal. SNP-Array analysis showed no copy number variants (CNVs) but identified loss of heterozygosity on chromosome 11. A clinical trio exome sequencing was performed on blood DNA from patient II:7 and both parents (I:1 and I:2) using the Agilent Clinical Research Exome V2 with sequencing on the Illumina Nextseq500 platform (Illumina, San Diego, CA, USA) which also came out negative.

Two years later a second affected sibling (II:8) was born. Given the previously negative genetic testing, parental consanguinity, and similar phenotype in the two affected siblings, we performed a whole-genome sequencing (WGS) in sibling II:8. The WGS was performed on genomic DNA extracted from a skeletal muscle biopsy using the Illumina TruSeq PCRfree (Illumina, San Diego, CA), with sequencing on the Illumina NovaSeq 6000 platform (Illumina). Sequence reads were aligned to the reference genome (hg19) and variant calling for single-nucleotide variants and small indels was performed using Sentieon DNAscope and Canvas for CNVs.

By including variants identified in the previously trio exome sequencing in individuals I:1, I:2 and II:7 together with variants identified by WGS performed in II:8 we selected variants that were present in both patients for further analysis. Detailed description for variant prioritization is found in the supplementary file.

### Muscle biopsy

Skeletal muscle biopsy was performed in II:8 at age 5 months for histopathology and mitochondrial functional analyses. For histopathology the specimen was snap-frozen in liquid propane chilled with liquid nitrogen for cryostat sectioning and histochemistry, and fixed in buffered glutaraldehyde for electron microscopy. Standard techniques were used for enzyme histochemistry, immunohistochemistry and electron microscopy [10]. Investigations of the respiratory chain function by oximetry and spectrophotometry in muscle mitochondria were performed as described [11].

### cDNA analysis on muscle specimen

For *RNH1* expression analysis, total RNA was isolated from fresh-frozen skeletal muscle of patient II:8 using the RNeasy Fibrous Tissue Mini Kit (Qiagen, Valencia, CA). RNA was reverse transcribed with the QuantiTect reverse transcription kit (Qiagen), and cDNA was analyzed by PCR and Sanger sequencing. The forward and reverse primers were designed to hybridize to different exons that were separated by large introns to generate a specific PCR-product on cDNA (detailed description is found in the supplementary file).

### Western blot analysis of RNH1 in muscle and fibroblasts

Western blot analysis of RNH1 was performed on protein extracted from sections of fresh frozen muscle biopsy specimens from patient II:8 and fibroblasts (detailed description is found in the supplementary file).

### RNA sequencing performed from fibroblasts

Fibroblasts derived from patient II:8 and fibroblasts derived from a control subject were grown and cells were transduced with control (empty vector) or RNH1 rescue lentivirus. Two days later, RNA was extracted and used for paired-end transcriptome sequencing. Low-quality reads were removed using SOAP [12] and the remaining reads were aligned to the reference genome GRCh38.p12) using BowTie2 [13]. Differentially expressed genes were calculated using DESeq2 (v1.4.5) [14]. Volcano plot and sequence coverage randomness were analyzed using the Dr. Tom Multi-omics Data mining system (https://biosys.bgi.com). Sashimi plots were generated using the Integrative Genome Viewer (IGV; v. 2.12.3).

### Lentiviral production

A cDNA clone encoding human full-length *RNH1* fused to a C-terminal V5-tag in the vector pLX304 (HsCD00442430) was obtained from the DNASU Plasmid Repository at Arizona State University. Lentivirus were produced in HEK293/T17 cells (ATCC, CRL-11268) grown in DMEM-GlutaMAX (Thermo Fisher Scientific) supplemented with 10% fetal bovine serum (FBS; Sigma-Aldrich). Cells were co-transfected with either control (empty) or *RNH1*-expressing lentiviral vectors together with 3rd generation lentiviral packaging plasmids using calcium-phosphate. Media was replaced 1 h prior to transfection to media supplemented with 25 mM chloroquine (Sigma-Aldrich) and replaced again 4–6 h later to medium without chloroquine. Conditioned media containing lentiviral particles were harvested 36–40 h later, cleared by centrifugation at 1500 × *g* for 10 min and snap-frozen in aliquots until use.

### RNase A tolerance assay

Fibroblasts from control and patient II:8 subjects were grown in DMEM-GlutaMAX containing 10% FBS. Cells were plated at a density of 100,000 cells per well of a 6-well plate and 24 h later transduced with control (empty vector) or RNH1 rescue lentivirus by adding 1/10 (vol/vol) of lentiviral particles. After 48 h, transduced cells were replated in flat-bottom 96-well plates at a density of 3500 cells/well for cell viability assay or on coverslips in 24-well plates at a density of 25,000 cells/well for cell death assay. Next day, cells were exposed to increasing concentrations of recombinant RNase A (Merck, #10109169001) in serum-free media (as bovine serum contains RNases). After 24 h, cell-viability was measured using the CellTiter 96 R Non-Radioactive Cell Proliferation Assay (MTT; Promega, #G4000) kit by replacing the media with 75 μl/well of serum-free media supplemented with 15 μl MTT dye (yellow tetrazodium dye). After 4 h of incubation at 37 °C, 100 μl of MTT stop solution was added to each well to solubilize the formazan product. Resulting absorbance at 570 nm was measured using a plate reader after overnight incubation at 4 °C. All conditions were included on each plate, which were normalized between experiments. To count apoptotic nuclei, cells subjected to 24 h of 30 μM RNAse treatment were fixed without prior washes with 4% paraformaldehyde, 4% sucrose in 0.1 M PBS for 15 min at room temperature and stained with Hoechst (Thermo Fischer Scientific, #33342, 1:5000) and Phalloidin-Atto 565 (Sigma, #94072). The coverslips were rinsed in distilled water and mounted with Prolong Gold Anti-Fade mounting medium (Thermo Fischer Scientific, P36934). Images were acquired using an A1plus confocal system (Nikon Instruments) with 20× NA1.0 objective. The images were analyzed using ImageJ (v. 2.9.0) and the number of apoptotic nuclei, defined as Hoechst-intense objects with a size of <77 μm^2^, were counted as proportion of the total number of nuclei per field.

### Statistics

Data were analyzed using Prism (v. 9.4.0; GraphPad). Statistical comparisons were performed using one-way ANOVA with Bonferroni correction, or two-way ANOVA with Holm-Sidak, as indicated in the figure legends.

## Results

### Case descriptions

**Fig. 1:**
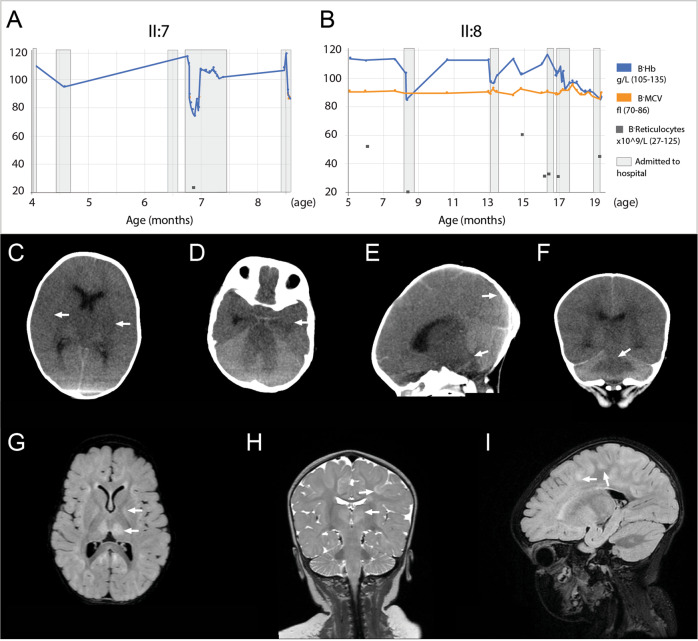
Hematological and neuroradiological investigations. **A**, **B** Both siblings developed macrocytic anemia (B-MCV > 86) with worsening during episodes of infections associated with levels of reticulocytes in the lower part of the normal level range suggestive of impaired erythropoiesis. In patient II:8, the baseline hemoglobin level tended to decrease over time. B-Hb = Blood-Hemoglobin, B-MCV = Blood-Mean Corpuscular Volume. Normal range of B-Hb, B-MCV and reticulocytes are given within parenthesis. **C**–**F** CT of the brain in patient II:7 at 8 months of age showing reduced attenuation of the entire supratentorial gray matter, compressed lateral ventricles and flattened extracerebral CSF spaces compatible with edema, loss of gray and white matter differentiation suggestive of ischemia and herniation (**E**, **F**) through the tentorium and foramen magnum. **G**–**I** In patient II:8 MRI of the brain at 8 months of age showed symmetrically increased T2 signal in thalamus, globus pallidus and in patchy cortical/subcortical fronto-parietal, paramedian and occipital areas.

We describe a family of seven children born to healthy first-cousin parents of Somali origin. Two siblings, one boy (II:7) and one girl (II:8), were affected by a disease characterized by congenital cataracts, global developmental delay, myopathy and infection-induced episodes of psychomotor deterioration, seizures, and macrocytic anemia. The mother had also previously lost a baby at week 20 of gestation. Detailed clinical descriptions for both patients follow, with principal hematological data provided in Fig. 1 (Fig. A, B) and detailed laboratory investigations given in Supplementary Table 1.

#### Patient II:7

This pregnancy was uncomplicated with delivery after 38 weeks of gestation. The birth weight was 3320 g (±0 SD), length 47 cm (−1.5 SD), head circumference 35.5 cm (+0.5 SD) and APGAR scores 9-10-10. Clinical examination revealed bilateral cataracts, which was surgically corrected at 2 months of age. At 5 days of age, he was admitted to the hospital because of sucking and feeding problems with a weight loss of 13%. He was listless with sparse movements, incomplete Moro reflex and muscular hypotonia. Because he was icteric, he received phototherapy for 12 h. He also received a glucose infusion followed by gavage feeding over 4 days. He was discharged after 10 days. Growth was normal and he had no malformations.

The global development was delayed. At 3 months of age, he gave eye contact, had social smile and adequate babble but was unstable in his neck control and showed pronounced head lag upon traction test. At 8 months of age, he displayed muscular hypotonia and weakness with normal tendon reflexes. He gave eye contact and responsive smiles. When put on his back, he grasped clumsily with his hands and lifted his lower arms with difficulty against gravity. In the prone position, he could lift his chest 45–90° with weight on his forearms and he could roll from back to prone position. On the traction test, there was head lag and extended arms, he needed hand support to sit and could bear weight on his legs upon standing.

From 4 months of age until his death at 8 months of age he had five hospital admissions due to deterioration related to viral upper respiratory tract infections with fever associated with breathing and feeding difficulties and weight loss. On these occasions, he was given oxygen, inhalations with adrenaline, salbutamol and NaCl and gavage feeding. These episodes were associated with anemia (Fig. 1A), without signs of bleeding or hemolysis.

On the third of these occasions, at 7 months of age, he additionally developed occasional focal seizures, a generalized tonic-clonic seizure lasting 3–5 min, as well as respiratory insufficiency that required ventilator treatment. EEG was normal. He had increased cerebrospinal (CSF)-levels of Neurofilament light (NFL) to 1760 ng/L (ref <380), Glial fibrillary acidic protein to 168,000 ng/L (ref <450), Total Tau (T-Tau) to 1370 ng/L (ref newborn <1000–1500, >1 year-olds <300) and Albumin to 334 mg/L (ref <225). Sleep studies revealed obstructive sleep apnoea due to hypotonia and he received a neck collar and VPAP home ventilation.

At 8 months of age, he developed fever, frequent vomiting and signs of dehydration. He became listless, more hypotonic and was admitted for intravenous rehydration. At hospital he developed a secondary generalized seizure and repeated multifocal seizures that were treated with diazepam. During an episode of tonic seizures and apnoea, he received ventilator treatment and was transferred to the intensive care unit (ICU). He was hemodynamically unstable and needed support by inotropic drugs. He became comatose without spontaneous respiration or reaction to pain. On the following day, a CT of the brain showed generalized supratentorial ischemia and cerebral herniation (Fig. 1C–F). He was diagnosed as brain dead, and the ventilator treatment was terminated.

#### Patient II:8

This 17-month-old female was the younger sibling of patient II:7. Pregnancy and delivery were normal. She was born after 41 + 2 weeks of gestation. The birth weight was 3370 g (−0.5 SD), length 48 cm (−1 SD), head circumference 36 cm (+0.8 SD) and APGAR scores 9-10-10. Ophthalmological investigations at an age of 2 weeks revealed bilateral exophoria and bilateral cataracts, the latter was operated at 5 weeks of age. Growth was normal and she had no malformations.

The global development was delayed. At 1 and 3 months of age, clinical examinations revealed a suspicious neck hypotonia. At 5 months of age, she had multi-syllable babbling while clinical examination showed a tent-shaped mouth, a small chin and bilateral exophoria. The patellar tendon reflexes were normal. There was muscular hypotonia with head lag and extended arms on a traction test and she was unable to fully balance her head in an upright position. She could partially take support on her legs in the standing position, lift her head with weight on her forearms in prone position and roll over from prone to supine position. She could grasp with both hands and move objects to her mouth. She had an increased CSF-level of NFL to 1530 ng/L (ref < 380). A brain MRI at 8 months of age showed symmetrically increased T2 signal in thalamus, globus pallidus and in patchy cortical/subcortical fronto-parietal, paramedian and occipital areas (Fig. 1G–I). Proton spectroscopy in basal ganglia and cerebral white matter was normal. At 1 year of age, she could laugh and say single words in Somali. She could sit for short periods without support and roll to one side but not ambulate. She was weak in her shoulders and arms and unable to extend her arms against gravity. She had bilateral pincer grasp and pulled herself actively to sitting on the traction test. At 17 months of age, she could walk with manual support, but developmental levels and neurological examinations were otherwise unchanged without signs of catch-up compared to her 1-year examination indicating developmental stagnation.

From 8 to 17 months of age, she has had five admissions to hospital because of deterioration related to viral infections with fever, obstructive breathing and feeding problems with vomiting and weight loss. During these infection-induced episodes, she became less responsive, more hypotonic and lost developmental skills. Moreover, she also developed intermittent anemia, initially with normalization in between episodes, but later with a tendency of the baseline hemoglobin (Hb) level to decline (Fig. 1B; for laboratory investigations, see Supplementary Table 1). A bone marrow exam taken in between episodes, showed normal cell distributions, no signs of dyserythropoeisis and, except for hypogranulation, no dysplasias in myelo- and megakaryopoeses. On the first episode she also had focal seizures, occasionally with secondary generalization, and developed respiratory insufficiency that required transient ventilator-treatment at the ICU. She was then also treated with oxygen, salbutamol, fluticasone propionate, gavage feeding, cefotaxime, dexamethasone, midazolam and received continuous treatment with levetiracetam. She then became stable in her epilepsy except for occasional short focal seizures during episodes of deterioration. At 1-year of age she received a gastrostomy for nutrition. She has also been treated with Q10, riboflavin and thiamine, from 5 months of age.

Muscle biopsy of the quadriceps muscle performed in patient II:8 at 5 months of age showed slight myopathic alterations with increased variability of fiber size, a few centrally located myonuclei and scattered regenerating muscle fibers (Supplementary Fig. 1A–C). No definite ultrastructural pathological changes were identified by electron microscopic analysis (Supplementary Fig. 1D, E).

### Biochemical mitochondrial investigations

Biochemical muscle mitochondrial investigations at 5 months of age showed unspecific changes with mildly reduced oxidation of complex I-related substrates, but with otherwise normal analyses including spectrophotometric measurements of the individual enzyme activities (for details see Supplementary Table 1).

### Molecular genetics

**Fig. 2:**
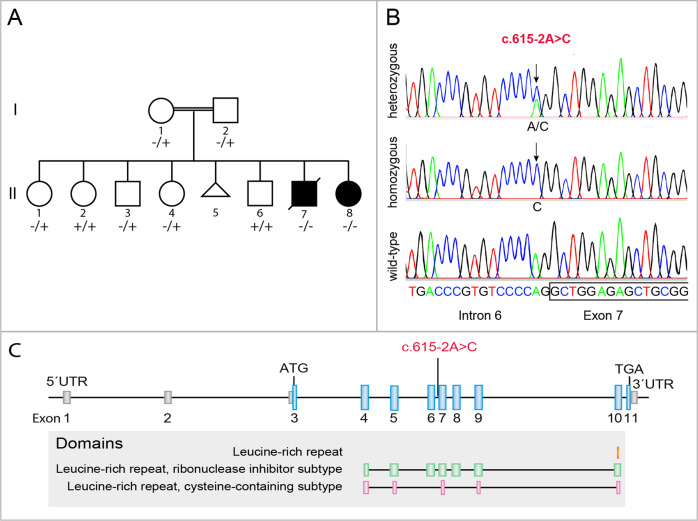
Pedigree and molecular genetics. **A** Pedigree of the family. Filled symbols indicate affected individuals. Segregation of the c.615-2A > C variant in the family is indicated by + (wild type) and – (variant). **B** Sequencing chromatograms showing the splice-site variant c.615-2A > C in *RNH1* indicated by an arrow. **C** Schematic illustration of the exon-intron structure of *RNH1* (NM_203387.3), including the novel splice-site variant identified in this study (in red, c.615-2A > C). Domains encoded by each exon are indicated.

The WGS together with the trio exome analysis revealed a rare homozygous splice-site variant (hg19 chr11: 498935 T > G reverse strand) in the ribonuclease/angiogenin inhibitor 1 gene (c.615-2A > C) (RNH1; OMIM: *173320, transcript NM_203387.3) in both affected siblings (II:7, II:8), which was not reported in any genome databases (i.e., ExAC, gnomAD, 1000 GP, dbSNP151, ESP). Homozygous *RNH1* variants were neither identified in several human knockout studies [15–20]. Further, segregation analysis in the family by Sanger sequencing showed that the homozygous variant segregated with disease in the family including 9 investigated individuals. The parents were heterozygous carriers (I:1, I:2) for the variant and the healthy siblings were either heterozygous carriers of the variant (II:1, II:3; II:4) or homozygous for the wildtype (II:2, II:6) (Fig. 2A, B). The variant was identified in the splice acceptor site of intron 6 (Fig. 2B, C) located in a homozygous region on chromosome 11 (chr11:≈196 000-7 620 000) (Supplementary Fig. 2). In silico splice site prediction algorithm MaxEntScan [21] predicts a complete loss in recognition of the canonical splice acceptor site in intron 6. A detailed description of the filtering criteria and other excluded variants are presented in the supplementary file. No large homozygous copy number variations were detected.

**Fig. 3:**
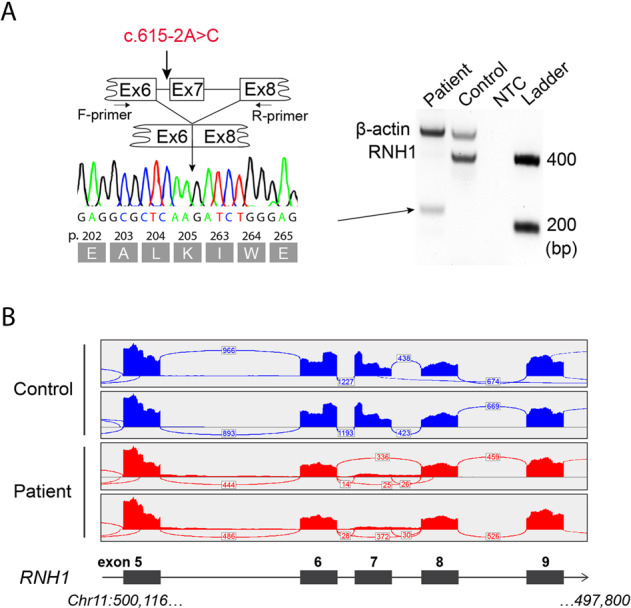
Analysis of aberrant RNH1 splicing in muscle and fibroblasts of patient II:8. **A** Schematic illustration showing the splice-site effect of the c.615-2A > C variant as investigated with reverse transcriptase polymerase chain reaction (RT-PC) followed by PCR and Sanger sequencing using forward primer located in exon 6 and reverse primer located in exon 8 on RNA extracted from muscle tissue (for forward primer located in exon 5 and reverse primer located in exon 9 see Supplementary Fig. 3A). In patient II:8 a band with lower molecular size was identified compared to a control sample using and Sanger sequencing chromatograms showing transcript lacking exon 7. **B** Sashimi-plots illustrating splicing of *RNH1* exons 5-9 (black boxes), generated from RNA-sequencing of patient-derived fibroblasts (II:8) (mock-transduced fibroblasts; Supplementary Fig. 5B). The numbers display junctions information for regions within the current IGV view. The “bridges” crossing exons indicate junction reads and the numbers of junction reads are shown on the “bridges”. Genome coordinates by GRCh38/hg38.

Further analysis of RNH1 cDNA from skeletal muscle of patient II:8 showed a PCR-product with lower molecular size compared to control samples. Sanger sequencing revealed a transcript lacking exon 7, which is predicted to result in an in-frame deletion of 57 amino acids (p.Leu206_Trp262del) (Fig. 3A and Supplementary Fig. 3A). The splice-defect was further investigated by analyzing *RNH1* reads obtained from RNA-sequencing of patient-derived fibroblasts (Fig. 3B). RNA-sequencing data confirm that exon 7 is skipped in nearly all reads.

RNH1 protein expression was studied by western blot analysis of proteins extracted from skeletal muscle in patient II:8, using both a monoclonal antibody raised against residues 1-135 of RNH1 and a polyclonal antibody (Supplementary Figs. 3B, 6, 7). These analyses revealed a band at the predicted size of 50 kDa in control samples, while patient samples showed a total absence of full-length protein. No detectable RNH1 protein of lower molecular weight corresponding to a product lacking exon 7 (calculated predicted molecular weight around 44 kDa) was detected. Data from the Genotype-Tissue Expression (GTEx) Portal Database (http://www.gtexportal.org) revealed that RNH1 is ubiquitously expressed across tissues (Supplementary Fig. 4). The constrain metrics of RNH1 gives a o/e score of 0.81 indicating that 20% less loss-of-function variants are observed than expected in the general population. This might indicate that biallelic loss-of-function variants are not well tolerated. In line with this, no homozygous carriers with loss-of-function variants in *RNH1* are listed in the gnomAD database (https://gnomad.broadinstitute.org/).

### Functional analysis in fibroblasts

**Fig. 4:**
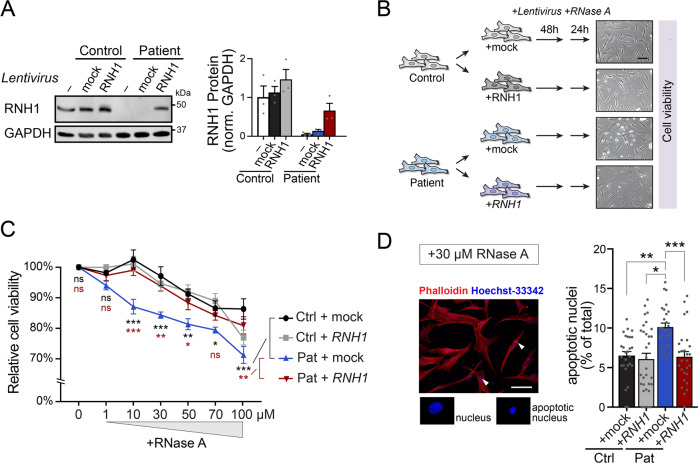
Functional investigations of patient-derived fibroblasts. **A** Western blot demonstrating lack of RNH1 protein in patient-derived fibroblasts and re-expression of RNH1-V5 by following lentiviral transduction. Data represented as mean ± standard error of the mean (s.e.m.; *n* = 3). **B** Outline of the RNase A tolerance assay. Inserts, DIC images of cells at time of analysis. Scale bar, 100 μm. **C** Relative cell viability following exposure to an increasing concentration of recombinant RNase A. Data represented as mean ± s.e.m. (*n* = 6). Statistical comparisons between (by brackets) indicated groups were done by two-way ANOVA with Holm-Sidak tests. *, *p* < 0.05; **, *p* < 0.01; ***, *p* < 0.001; ns, not significant. **D** Quantitative analysis of the proportion of apoptotic cell nuclei, detected by nuclear Hoechst-staining, after 24 h exposure to 30 μM RNase A. Representative phalloidin- and Hoechst-stained cells (left) with apoptotic nuclei indicated by arrowheads, and summary data (right), represented as mean ± s.e.m. (*n* = 25–28 fields of view from a total of four independent experiments). Scale bar, 100 μm. Statistical comparisons by one-way ANOVA with Bonferroni correction. *, *p* < 0.05; **, *p* < 0.01; ***, *p* < 0.001.

To address functional effects of the genetic variant, we used skin fibroblasts from patient II:8. The patient-derived fibroblasts showed similar growth rates as fibroblasts derived from a matched control, both under basal and serum-deprived conditions (Supplementary Fig. 5A). As RNH1 has been shown to confer protection against extracellular RNases [1, 22], we subjected fibroblasts from the patient and a matched control to an RNase A tolerance assay. As a rescue experiment, we transduced RNH1-deficient patient fibroblasts with a lentivirus expressing RNH1 to complement patient cells. Transduced cells expressed ~70% of RNH1 compared to control cells (Fig. 4A). Next, we exposed transduced cells to increasing concentrations of recombinant RNase A in the media, and measured cell viability 24 h later using the MTT assay (Fig. 4B). Patient cells were more sensitive to RNase exposure, demonstrating a ~15% lower viability at RNase A concentrations of 10 uM and higher (Fig. 4C). Importantly, this phenotype was fully reversed when *RNH1* was re-expressed (Fig. 4C). As the MTT assay cannot distinguish between reduced growth rates and increased cell death, we fixed and stained cells at the end of the experiment to count apoptotic nuclei. We found a significant increase in the proportion of apoptotic nuclei in non-complemented patient cells (Fig. 4D).

We performed transcriptome analysis by bulk RNA-sequencing of patient and control fibroblasts. Three conditions were compared: control fibroblasts transduced with control lentivirus, patient cells with control virus and, as a stringent isogenic control, patient cells complemented with *RNH1*-expressing virus (Supplementary Fig. 5B). Comparing patient cells to patient cells expressing *RNH1*, no differentially expressed genes were identified with genome-wide significance, except for the expected lentiviral RNH1 reads (Supplementary Fig. 5C and Supplementary Table 6). From this we conclude that *RNH1* does not regulate basal transcript levels, at least in fibroblasts.

## Discussion

Here we provide the first report of a human disease caused by a homozygous null variant in *RNH1*. We identified two affected siblings with a disease characterized by congenital cataracts, global developmental delay, myopathy, and infection-induced episodes of psychomotor deterioration, seizures, and macrocytic anemia. The identified homozygous variant segregated with disease in the family and has so far not been identified in reference genome databases such as gnomAD. The variant (c.615-2A > C) exerts a loss-of-function effect by altering the canonical splice acceptor site, which causes skipping of exon 7 and expression of a transcript lacking 57 codons, which encodes a part of the leucine-rich repeat domain. No aberrant spliced protein could be detected by western blot analysis, suggesting that the predicted mutant protein is degraded and that the patients are functionally ‘knockouts’ regarding RNH1.

Constitutive *Rnh1* knockout mice are lethal at embryonic day E8.5-E10 [9], suggesting a possible species-to-species difference compared to humans lacking *RNH1*, as shown in our study. *RNH1* knock-out mice were reported to die from anemia caused by failed erythroid cell maturation due to deficient translation of the transcription factor GATA1 [9]. It was therefore interesting to note that our patients displayed macrocytosis and developed anemia not only secondary to rehydration. As in Diamond-Blackfan anemia, caused by mutations in ribosomal protein genes [23], the macrocytic anemia in our patients was not accompanied by significant other cytopenias. In the patient (II:8) where bone marrow examination was performed, the erythropoiesis was normal without signs of dyserythropoesis or megaloblastic anemia. Nor were there any signs of dysmegakaryopoesis, which has been reported in the X-linked anemia caused by mutation in *GATA1* [24]. Based on the observed tendency of decreased Hb, it cannot be ruled out that over time a condition similar to the pure red cell aplasia seen in Diamond-Blackfan anemia might develop [25].

*RNH1* is ubiquitously expressed (the GTEx Portal Database; http://www.gtexportal.org) (Supplementary Fig. 4), suggesting a role in various tissues. Consistent with this notion, we could detect a cellular defect also in skin-derived fibroblasts. Patient fibroblasts, lacking RNH1, were more sensitive to extracellular RNase A than cells from a control subject or patient cells complemented by RNH1 expression. RNH1 is known to bind secreted ribonucleases, such as RNase1 (RNase A), RNase2 and RNase4 [1], which are important players in the innate immune defense by immunomodulatory and antimicrobial properties [26, 27]. RNases are secreted by cells such as immune cells, endothelial cells, epithelial cells and fibroblasts participating in the host defense against infection. They are found in all body fluids and their levels correlate with infection and inflammation processes. After secretion, the RNases may re-enter cells by endocytosis where they can cause severe damage and even be fatal to the cells unless the cells are protected by RNase inhibitors such as RNH1, which inactivates the RNase A by strong (femtomolar affinity) binding forming RNase-RNH1 complexes [22]. Our result supports a role for RNH1 in protecting against extracellular RNases and is in line with results from *RNH1* knockout HeLa cells [22]. The concentrations of RNase A that are cytotoxic in our assays are considerably higher than the nanomolar range reported in human serum [22]. However, cell types other than fibroblasts may be susceptible to lower levels of RNases. The levels of extracellular RNases may also increase significantly during infection and inflammation, which may explain why our patients’ disease progression appeared to correlate with common infections.

Nevertheless, we cannot exclude that loss of some of the many other proposed functions of *RNH1* may drive disease pathophysiology. For example, RNH1 has been proposed to regulate inflammation via inflammasomes, another component of the innate immune defense. Inflammasomes are cytosolic multiprotein complexes consisting of a sensor molecule, such as NLR-family pyrin domain-containing 3 (NLRP3), the adaptor protein ASC and cysteine protease pro-caspase-1 [28]. Activated inflammasomes promote, through activated caspase-1, release of pro-inflammatory cytokines and may induce pyroptosis, a form of lytic cell death [29]. Inflammasome activation, if not controlled, may thus be detrimental to the organism and may be involved in the tissue destruction observed in various inflammatory, autoimmune, autoinflammatory and age-related diseases [30, 31]. A recent study has shown that RNH1 can attenuate inflammasome activation and thereby protects the organism from uncontrolled inflammasome activation [6]. It can therefore be speculated that infection-induced deterioration in our patients may be associated with lack of RNH1-mediated control of inflammasome activation.

Both siblings had congenital cataracts as part of their disease manifestations. We therefore note with interest that RNases have been reported to be involved in the development of cataracts. In normal human and animal lenses, no RNase activity is detected but senile cataracts is accompanied by an increase in RNase activity [32]. Very high levels of RNase inhibitor in normal lenses explain the total absence of RNase activity [33], but these levels decreased profoundly with development of cataracts, both in senile cataracts in humans and in experimental cataracts in rats, concomitant with increased RNase activities [33]. It was hypothesized from these results that the development of cataracts is closely linked to loss of RNase inhibitor activity. In our patients this hypothesis may be relevant and related to the congenital cataracts associated with lack of RNH1, although the mechanisms remain elusive.

Both patients showed delayed motor development and muscle weakness indicating involvement of the neuromuscular system. Muscle biopsy in patient II:8 revealed myopathic changes including slightly increased variability of fiber size, some fibers with abnormal central localization of nuclei and ongoing muscle fiber regeneration revealed by expression of embryonic myosin heavy chain, which indicates preceding muscle fiber necrosis. These myopathic changes are mild and unspecific but indicate that the lack of RNH1 is harmful to muscle. The mechanism can only be speculated on, but presence of necrosis followed by regeneration may be due to episodic increase in levels of RNases endocytosed by the muscle cells and lack of protective inhibition by RNH1.

*RNH1* is expressed in the brain and RNase1 is normally present in CSF fluid [34], suggesting that RNH1 may be essential for normal homeostasis by protecting the brain against RNase exposure. The involvement of the central nervous system in the disease in our patients was indicated by clinical signs and symptoms including global developmental delay, psychomotor deterioration, seizures, brain MRI alterations and increased levels of the CSF markers NFL and GFAP suggestive of neuroaxonal and astroglial injury. One of the siblings died of brain edema during an episode of deterioration associated with fever. The episodic psychomotor deterioration associated with infections was suggestive of a neurometabolic disease, but screening for neurometabolic diseases by laboratory investigations including mitochondrial function did not disclose any such metabolic disease.

In conclusion, we describe two siblings with a homozygous loss-of-function variant in *RNH1* associated with a disorder characterized by congenital cataracts, global developmental delay, myopathy and infection-induced episodic psychomotor deterioration, seizures, and anemia. We provide functional evidence that lack of RNH1 in patient fibroblasts cause susceptibility toward extracellular RNases, which could explain the multiorgan manifestations and infection-induced deterioration.

## Supplementary information

Supplementary file

Supplementary table 6

## Data Availability

The datasets generated during the current study are available upon request.
