# Supplementary material for: Ribonuclease inhibitor 1 (RNH1) deficiency cause congenital cataracts and global developmental delay with infection-induced psychomotor regression and anemia
Source: Eur J Hum Genet. 2023 Mar 20;31(8):887–94. doi: 10.1038/s41431-023-01327-7 (PMC10400601; doi:10.1038/s41431-023-01327-7)
Supplement: Supplementary file 1 — Supplementary file [file 41431_2023_1327_MOESM1_ESM.docx]

**Supplementary material**

**Ribonuclease inhibitor 1 (*RNH1*) deficiency cause congenital cataracts and global developmental delay with infection-induced psychomotor regression and anemia**

Carola Hedberg-Oldfors^1^, Sanhita Mitra^1,2^, Angela Molinaro^1^, Kittichate Visuttijai^1^, Linda Fogelstrand^1,3^, Anders Oldfors^1^, Fredrik H. Sterky^1,2,3^ and Niklas Darin^4^

^1^ Department of Laboratory Medicine, Institute of Biomedicine, Sahlgrenska Academy at the University of Gothenburg, Gothenburg, Sweden

^2^ Wallenberg Centre for Molecular and Translational Medicine, University of Gothenburg, Gothenburg, Sweden

^3^ Department of Clinical Chemistry, Sahlgrenska University Hospital, Gothenburg, Sweden

^4^ Department of Pediatrics, University of Gothenburg, The Queen Silvia Children’s Hospital, Gothenburg, Sweden

*Corresponding author: Carola Hedberg-Oldfors; Department of Laboratory Medicine, University of Gothenburg, Gothenburg, Sweden. Address: Sahlgrenska University Hospital, Gula stråket 8, 413 45 Gothenburg, Sweden, phone number +46 31 342 2887, e-mail: carola.oldfors@gu.se.

**Material and methods**

**Genetic testing**

For variant prioritization we searched for variants based on the assumption of recessive inheritance e.g., homozygous or compound heterozygous variants satisfying the following criteria: i) population frequencies with a minor allele frequency (MAF) < 0.5% in NHLBI ESP exomes, ExAC or gnomAD (gnomAD 2.1.1), ii) affecting protein-coding or splice-site regions, iii) predicted to be possibly deleterious/damaging using different *in silico* tools such as CADD (CADD score >17) (version v1.6) (1), PolyPhen-2 (v2.2.2) (2), BSIFT (2016-02-23) (3), Clinvar (2020-09-15), MAxEndScan (4) and HGMD (2021.1). REVEL (5) and M-CAP (6) were further used to for pathogenicity prediction of identified missense variants. Variants with high allele frequency in the African/African American population or identified as homozygous in the gnomAD database was excluded for further analysis. Genes and variants were further curated to be able to exclude variants not likely to be associated with the disease based on the clinical information (a detailed description on the filtering criteria is included in the supplementary material, Table S2-5). Candidate variants were confirmed by Sanger sequencing and segregation studies were performed with available DNA samples.

**cDNA analysis on muscle specimen**

For *RNH1* expression analysis, total RNA was isolated from fresh-frozen skeletal muscle of patient II:8 using the RNeasy Fibrous Tissue Mini Kit (Qiagen, Valencia, CA). RNA was reverse transcribed with the QuantiTect reverse transcription kit (Qiagen), and cDNA was analyzed by PCR and Sanger sequencing. The forward and reverse primers were designed to hybridize to different exons that were separated by large introns to generate a specific PCR-product on (two set of primer pairs were used; 1; forward primer located in exon 6 and reverse primer located in exon 8, 2; forward primer located in exon 5 and reverse primer located in exon 9). Primer sequences and PCR conditions are available upon request. The β-actin gene (NM_001101.3) was used as an internal control.

**Western blot analysis of RNH1 in muscle and fibroblasts**

Western blot analysis of RNH1 was performed on protein extracted from sections of fresh frozen muscle biopsy specimens from patient II:8. Cleared lysates were loaded and separated on a 4‒12 % Bis-Tris-Protein gel (Novex NP0322BOX; Thermo Fisher Scientific), followed by electroblotting. The membranes were incubated either with a monoclonal antibody (A-9; Santa Cruz Biotechnology, Dallas, TX, sc-365783; used at 1:500 dilution) or a polyclonal antibody (Abcam H00006050-B01P; used at 1:500 dilution) against human RNH1 as primary antibodies. The SuperSignal West Femto Maximum Sensitivity Substrate (Thermo Fisher Scientific) was used for antibody detection. The band corresponding to myosin heavy chain in the Coomassie-stained gel was used as loading control. For analysis in fibroblasts, cells plated at a density of 100,000 cells per well in a 6-well plate were transduced 24h after plating with 1/10 (vol/vol) of control (empty vector) or RNH1-expressing lentivirus. Protein lysates were collected after 48h after transduction, separated on a 4-20% Criterion TGX gels (Bio-Rad), and transferred to a PVDF membrane. Antibodies against RNH1 (see above) or GADPH (ab8245, Abcam; used at 1:5000). Near-infrared 680RD- or 800CW-coupled secondary antibodies (LI-COR) were used for detection. Membranes were scanned using an Odyssey CLx System (LI-COR) and band densities quantified using Image Studio Lite software (v. 5.2.5).

**RNA sequencing performed from fibroblasts**

Fibroblasts derived from patient II:8 and fibroblasts derived from a control subject were grown in DMEM-GlutaMAX (Thermo Fisher Scientific, Waltham, MA) supplemented with 10% FBS (Hyclone; Cytiva Marlborough, MA). Cells were transduced with control (empty vector) or RNH1 rescue lentivirus by adding a 10% (vol/vol) of virus-conditioned supernatant. Two days later, RNA was extracted using the ReliaPrep RNA Cell Miniprep System (Promega, Madison, WI) and used for paired-end transcriptome sequencing performed on a BGISEQ platform (BGI Europe, Copenhagen, Denmark). Sequencing data were filtered from adaptor sequences, low-quality reads (containing >20% of bases with quality ≤15 or ‘N’ base ratio >5% using SOAP (7). Reads were aligned to the reference genome dataset GCF_000001405.38_GRCh38.p12 (BGI) using BowTie2 (8). Differentially expressed genes were calculated using DESeq2 (v1.4.5) (9). Volcano plot and sequence coverage randomness were analyzed using the Dr. Tom Multi-omics Data mining system (https://biosys.bgi.com). Sashimi plots were generated using the Integrative Genome Viewer (IGV; v. 2.12.3).

**Supplementary Table 1. Results from laboratory investigations**

|  | Patient II:7 | Patient II:8 |
| --- | --- | --- |
| Haematological investigations | | |
| B-Haemaglobin (g/L) minimum level | **71** (95-135) | **86** (105-135) |
| B-EVF minimum level | **0.220** (0.290-0.420) | **0.250** (0.290-0.420) |
| B-Reticulocyte count (x 10x^9^) | 25 (20-100) | 28-40 (27-125) |
| B-MCV maximum level | **89** (70-86) | **91** (70-86) |
| B-Erythrorcytes (x 10x^12^)- minimum level | **3.14** (3.7-5.3) | **3.22** (3.7-5.3) |
| B-Leukocytes (x 10x^9^) | **5.3**-11.3 (6-17.5) | **5**-7.7 (6-17.5) |
| B-Neutrophils (x 10x^9^) | 2.3-5.7 (1-8.5) | **0.92**-3.2 (1-8.5) |
| B-Monocytes (x 10x^9^) | 0.36-0.7 (0.1-1.0) | 0.36-**1.2** (0.2-0.8) |
| B-Lymphocytes (x 10x^9^) | **1.8** (4-13.5) - 2.13 (1.1-4.4) | **3.3**-5.4 (4-13.5) |
| B-Thrombocytes (x 10x^9^) | 272-**437** (170-400) | 295-**648** (210-590) |
| P-Bilirubin (μmol/L) | < 3 (5-25) | 2-4.7 (< 25) |
| S-Haptoglobin (g/L) | ND | 1.4-**3.9** (< 2.9) |
| S-LD (μkat/L) | ND | **5.2**-**5.7** (2.2-4.9) |
| Morphological bone marrow examination | ND | Normal except for **hypogranulation of neutrophilic cells** |
| Normal investigations |  | P-iron, P-transferrin, P-ferritin, P-cobalamin, P-folate, B-erythrocyte morphology, B-hemoglobin electrophoresis, S-erythropoietin, e-ADA |
| CSF-analyses | At 7 m. | At 5 m. |
| Albumin (mg/L) | **334** (<225) | 149 (<225) |
| GFAp (ng/L) | **168 000** (< 450) | 220 (< 450) |
| T-Tau (ng/L) | **1370** (newborn < 1000-1500, > 1 y < 300) | 770 (newborn <1000-1500, > 1 y < 300) |
| NFL (ng/L) | **1760** (< 380) | **1 530** (< 380) |
| Metabolic investigations with normal results | | |
|  | P-thyroid hormones, P/U amino acids and U-organic acids, acyl carnitines, and peroxisomal screening. | P-lactate and -pyruvate, P-ammonia, S-copper, S-ceruloplasmin, creatine kinase, P/U-amino acids, S-acyl carnitines, S-CDT, S-biotinidase, P-peroxisomal screening, U-organic acids, U-glycoconjugates. Biochemical muscle mitochondrial investigations at 5 m. showed mildly reduced oxidation of complex I-related substrates and normal spectrophotometric enzyme activities. |
| Immunological investigations with normal results | | |
|  |  | CRP, SR, S- calprotectin, Blood T-cell stimulation, S- immunoglobins, IgG-subclasses, S-cytokines (IL-1beta, IL-6, IFN-gamma, TNF-α soluble IL-2-receptor, IL-18) were normal. B-inborn immunity panel after stimulation of toll-like receptor -2, -3, -4 showed normal production of TNF-α, IL-10 and IL-12. |
| Miscellaneous investigations with normal results | | |
|  | Routine serum biochemistry, S-TORCH, PCR-CMV  Chromosomal karyotyping, SNP-Array analysis, *SMA*, *PWS* and *DM1* | Routine serum biochemistry, S-TORCH |
| Cardiological investigations (EKG and UCG) | At 2 w. and 8 m. normal | At 8 m. normal |
| EEG | At 7 m. normal | At 5 and 8 m. normal |
| Neuroimaging | At 2 w. MRI normal  At 8 m. CT, see Fig 1 | At 1m. MRI normal  At 8 m. MRI, see Fig 1 |

**Abbreviations:** Minimum and maximum levels are given when analyses have been repeated. Abnormal results are given in bold font. Reference values are given inside the parentheses. Abbreviations are given in alphabetical order: CSF= cerebrospinal fluid, CT= computed tomography, DM1= myotonic dystrophy type 1, EEG= Electroencephalogram, EKG= Electrocardiogram, EVF= erythrocyte volume fraction, GFAp= glial fibrillary acidic protein, LD= lactate dehydrogenase, m.= months, MCV= mean corpuscular volume, MRI= magnetic resonance imaging, ND= not done, NFL= neurofilament light, PWS= Prader-Willi syndrome, S-TORCH= newborn infection screen for toxoplasma, rubella, cytomegalovirus and herpes simplex, SMA= spinal muscular atrophy, T-Tau= total tau, UCG= echocardiogram, w.= weeks, y.= years.

**Supplementary Table 2.** Description on the filtering criteria for identified homozygous variants

| Filtering | Combination of WGS and ES | II:7 and II:8 |
| --- | --- | --- |
| 1 | Total variants | 5 444 209 |
| 2 | Confidence | 5 434 487 |
| 3 | Common Variants <0.5% | 456 173 |
| 4 | Genetic analysis (homozygous) | 294 |
| 5 | Predicted Deleterious | 110 |
| 6 | Manually curated | 24 |
| 7 | Candidate variant | 1 |

WGS, whole-genome sequencing; ES, exome sequencing

Filter Description:

1) Starting with 5 444 209 variants spanning 30,880 genes, variants were:

2) Kept variants with call quality of at least 7.0 in cases

3) Excluded variants that are observed with an allele frequency greater than or equal to 0.5% of the genomes in the 1000 genomes project OR greater than or equal to 0.5% of the NHLBI ESP exomes (All) OR greater than or equal to 0.5% of the ExAC Frequency OR greater than or equal to 0.5% of the gnomAD Frequency UNLESS established Pathogenic common variant

4) Kept variants which are homozygous AND occur in at least 2 of the case samples at the variant level in the Case samples AND not which are heterozygous

5) Kept variants that are experimentally observed to be associated with a phenotype: Pathogenic, Possibly Pathogenic, Unknown Significance OR Disease-associated according to HGMD OR Frameshift, in-frame indel, or stop codon change OR Missense OR predicted deleterious by having CADD score > 17.0 OR splice site within 10 bases into intron OR predicted to disrupt splicing by MaxEntScan

6) Considering parents to be heterozygous, true in IGV and removing false positive

# see **Supplementary Table 4**

7) Keeping variant after considering patients phenotype and literature search (marked red in Supplementary Table 4)

**Supplementary Table 3.** Description on the filtering criteria for identified heterozygous variants

|  | Combination of WGS and ES | II:7 and II:8 |
| --- | --- | --- |
| 1 | Total variants | 5 444 209 |
| 2 | Confidence | 5 434 487 |
| 3 | Common Variants <0.5% | 456 173 |
| 4 | Genetic analysis (heterozygous) | 2855 |
| 5 | Predicted Deleterious | 2371 |
| 6 | Genetic analysis (at least two heterozygous variants within a gene) | 91 |
| 7 | Genetic analysis (compound heterozygous variants) | 14# |
| 8 | Manually curated | 0 |
| 9 | Candidate variant | 0 |

WGS, whole-genome sequencing; ES, exome sequencing

Filter Description:

1) Starting with 5 444 209 variants spanning 30,880 genes, variants were:

2) Kept variants with call quality of at least 7.0 in cases

3) Excluded variants that are observed with an allele frequency greater than or equal to 0.5% of the genomes in the 1000 genomes project OR greater than or equal to 0.5% of the NHLBI ESP exomes (All) OR greater than or equal to 0.5% of the ExAC Frequency OR greater than or equal to 0.5% of the gnomAD Frequency UNLESS established Pathogenic common variant

4) Kept variants which are heterozygous AND occur in at least 2 of the case samples at the variant level in the Case samples AND not which are homozygous in the Cases or gnomAD

5) Kept variants that are experimentally observed to be associated with a phenotype: Pathogenic, Possibly Pathogenic, Unknown Significance OR Disease-associated according to HGMD OR Frameshift, in-frame indel, or stop codon change OR Missense OR predicted deleterious by having CADD score > 17.0 OR splice site within 10 bases into intron OR predicted to disrupt splicing by MaxEntScan

6) Both Cases have the same two heterozygous variants

7) Compound heterozygous variants

# see **Supplementary Table 5**

8) Variant after considering patients phenotype and literature search.

**Supplementary Table 4.** Identified variants homozygous in both patients and heterozygous in the parents

| Gene | Transcript ID | Transcript | Protein | Translation | Classif- | SIFT | SIFT | PolyPhen-2 | M-CAP | M-CAP | CADD | REVEL | Conservation | MaxEnt | gnomAD | gnomAD |
| --- | --- | --- | --- | --- | --- | --- | --- | --- | --- | --- | --- | --- | --- | --- | --- | --- |
| Symbol |  | Variant | Variant | Impact | ication | Pred. | Score | Pred. | Score | Pred. | Score |  | phyloP p-value | Pred. | Frequency | African |
| MORN1 | NM_024848.3 | c.468C>T | p.D156D | synonymous | VUS |  |  |  |  |  | < 10 |  | 1.991E-3 | 0.0% | 0.008 | 10/21536 |
| ARL13B | NM_001174150.2 | c.130+7T>C |  |  | VUS |  |  |  |  |  | < 10 |  |  | NI | 0.019 | 48/24822 |
| OR5K1 | NM_001004736.4 | c.233C>A | p.T78N | missense | VUS | D | 0.01 | B | 0.003 | LB | < 10 | 0.030 |  | NI | 0.004 | 9/24952 |
| NCEH1 | NM_020792.6 | c.853C>T | p.R285C | missense | VUS | T | 0.11 | PD | 0.035 | PP | 22.800 | 0.324 |  | NI |  | - |
| FSTL4 | NM_015082.2 | c.2271G>A | p.T757T | synonymous | VUS |  |  |  |  |  | < 10 |  |  | NI | 0.001 | 0/24964 |
| HPS1 | NM_000195.5 | c.565C>T | p.R189W | missense | VUS | D | 0.00 | PD | 0.091 | PP | 28.500 | 0.184 | 2.415E-4 | NI | 0.001 | 0/24912 |
| MRPL43 | NM_176794.2 | c.422G>A | p.R141H | missense | VUS | T | 0.54 | B | 0.007 | LB | 13.360 | 0.033 |  | NI | 0.012 | 31/24942 |
| LZTS2 | NM_032429.4 | c.222C>T | p.C74C | synonymous | VUS |  |  |  |  |  | 12.560 |  | 5.598E-5 | NI | 0.016 | 36/24832 |
| NPM3 | NM_006993.3 | c.435T>A | p.D145E | missense | VUS | T | 0.48 | B | 0.005 | LB | 12.970 | 0.038 | 1.923E-4 | 0.0% | 0.034 | 89/24952 |
| NOLC1 | NM_004741.5 | c.1849-8T>G |  |  | VUS |  |  |  |  |  | 13.100 |  | 3.776E-4 | -6.1% | - | - |
| GBF1 | NM_004193.3 | c.2104-6A>T |  |  | VUS |  |  |  |  |  | < 10 |  |  | +36.0% | 0.021 | 46/24898 |
| ACTR1A | NM_005736.4 | c.132C>T | p.H44H | synonymous | VUS |  |  |  |  |  | < 10 |  | 2.009E-8 | NI | 0.017 | 26/23104 |
| RNH1 | NM_203387.3 | c.615-2A>C |  | Splice Site Loss | VUS |  |  |  |  |  | 32.000 |  |  | -100.0% | - | - |
| EPS8L2 | NM_022772.4 | c.1060-6C>T |  |  | VUS |  |  |  |  |  | < 10 |  |  | +3.2% | 0.033 | 49/24914 |
| TALDO1 | NM_006755.2 | c.181C>G | p.L61V | missense | VUS | D | 0.02 | B | 0.092 | PP | 22.600 | 0.568 | 3.565E-8 | NI | 0.034 | 52/24912 |
| PIDD1 | NM_145887.4 | c.1917+10C>T |  |  | VUS |  |  |  |  |  | < 10 |  |  | NI | 0.041 | 38/16284 |
| ART5 | NM_053017.5 | c.639C>T | p.F213F | synonymous | VUS |  |  |  |  |  | < 10 |  | 3.034E-6 | NI | 0.025 | 26/24968 |
| RHOG | NM_001665.4 | c.405G>A | p.A135A | synonymous | VUS |  |  |  |  |  | 10.510 |  | 4.159E-3 | NI | 0.002 | 0/24902 |
| OR56B1 | NM_001005180.3 | c.557G>C | p.C186S | missense | VUS | T | 0.31 | PD | 0.018 | LB | 23.300 | 0.229 |  | NI | - | - |
| OR52N4 | NM_001005175.5 | c.711G>A | p.Q237Q | synonymous | VUS |  |  |  |  |  | < 10 |  | 8.650E-10 | NI | - | - |
| TEAD1 | NM_021961.6 | c.252G>A | p.T84T | synonymous | VUS |  |  |  |  |  | 12.540 |  | 2.600E-4 | NI | 0.004 | 6/24944 |
| PTH | NM_000315.4 | c.230C>A | p.A77D | missense | LB | T | 0.06 | B | 0.041 | PP | < 10 | 0.125 |  | NI | 0.007 | 1/16256 |
| FAM117A | NM_030802.4 | c.505C>T | p.R169C | missense | VUS | D | 0.00 | PD | 0.028 | PP | 25.700 | 0.183 | 2.173E-3 | NI | 0.004 | 0/16222 |
| ABCA5 | NM_172232.4 | c.2449G>T | p.D817Y | missense | VUS | D | 0.05 | PD | 0.098 | PP | 28.200 | 0.636 |  | NI | 0.044 | 86/23520 |

VUS, Uncertain Significance; D, damaging; T, tolerated; B, benign; PD, possible/probably damaging; LB, Likely benign; PP, Possibly Pathogenic; NI, no impact; Pred, prediction

**Supplementary Table 5.** Identified variants compound heterozygous in both patients and heterozygous in the parents

| Gene | Transcript ID | Transcript | Protein | Translation | Classif- | SIFT | SIFT | PolyPhen-2 | M-CAP | M-CAP | CADD | REVEL | Conservation | MaxEnd | gnomAD | gnomAD |
| --- | --- | --- | --- | --- | --- | --- | --- | --- | --- | --- | --- | --- | --- | --- | --- | --- |
| Symbol |  | Variant | Variant | Impact | ication | Pred. | Score | Pred. | Score | Pred. | Score |  | phyloP p-value | Prediction | Frequency | African |
| DLGAP2 | NM_001346810.2 | c.1040A>G | p.N347S | missense | VUS |  |  |  | 0.004 | LB | < 10 | 0.120 |  | NI | 0.008 | 1/23882 |
| DLGAP2 | NM_001346810.2 | c.3162G>A | p.R1054R | synonymous | VUS |  |  |  | - |  | 12.610 |  | 4.111E-8 | NI | 0.006 | 0/19444 |
| CHTF18 | NM_022092.3 | c.2478C>T | p.R826R | synonymous | VUS |  |  |  | - |  | < 10 |  | 3.119E-8 | NI | 0.001 | 0/23744 |
| CHTF18 | NM_022092.3 | c.2581C>T | p.R861W | missense | VUS | T | 0.06 | B | 0.012 | LB | 11.090 | 0.040 |  | NI | 0.004 | 4/23718 |
| PKD1 | NM_000296.4 | c.8541G>A | p.K2847K | synonymous | VUS |  |  |  |  |  | < 10 |  | 3.784E-8 | NI | - | - |
| PKD1 | NM_000296.4 | c.8136C>G | p.I2712M | missense | VUS |  |  | PD | 0.903 | PP | < 10 | 0.319 | 2.897E-9 | NI | - | - |
| PKD1 | NM_000296.4 | c.5394G>A | p.V1798V | synonymous | VUS |  |  |  |  |  | < 10 |  | 2.938E-9 | NI | - | - |
| MTSS2 | NM_138383.3 | c.2095C>G | p.L699V | missense | VUS | T | 0.56 | B | 0.024 | LB | 15.420 | 0.060 |  | NI | - | - |
| MTSS2 | NM_138383.3 | c.884G>T | p.G295V | missense | VUS | D | 0.01 | PD | 0.040 | PP | 25.800 | 0.125 | 4.786E-4 | NI | - | - |
| RHPN2 | NM_033103.5 | c.1479G>A | p.T493T | synonymous | VUS |  |  |  |  |  | < 10 |  |  | NI | 0.033 | 5/24966 |
| RHPN2 | NM_033103.5 | c.199T>C | p.S67P | missense | VUS | T | 0.36 | B | 0.003 | LB | 15.510 | 0.054 | 7.889E-6 | NI | 0.000 | 0/16162 |
| PTPRH | NM_002842.5 | c.3035A>C | p.E1012A | missense | VUS | T | 0.73 | B | 0.055 | PP | 11.260 | 0.136 |  | NI | 0.044 | 117/24942 |
| PTPRH | NM_002842.5 | c.2257+16C>A |  |  | VUS |  |  |  |  |  | < 10 |  |  | NI | - | - |
| PTPRH | NM_002842.5 | c.227C>T | p.T76I | missense | VUS | T | 0.06 | B | 0.015 | LB | < 10 | 0.111 |  | NI | 0.010 | 27/24958 |

VUS, Uncertain Significance; D, damaging; T, tolerated; B, benign; PD, possible/probably damaging; LB, Likely benign; PP, Possibly Pathogenic; NI, no impact; Pred, prediction


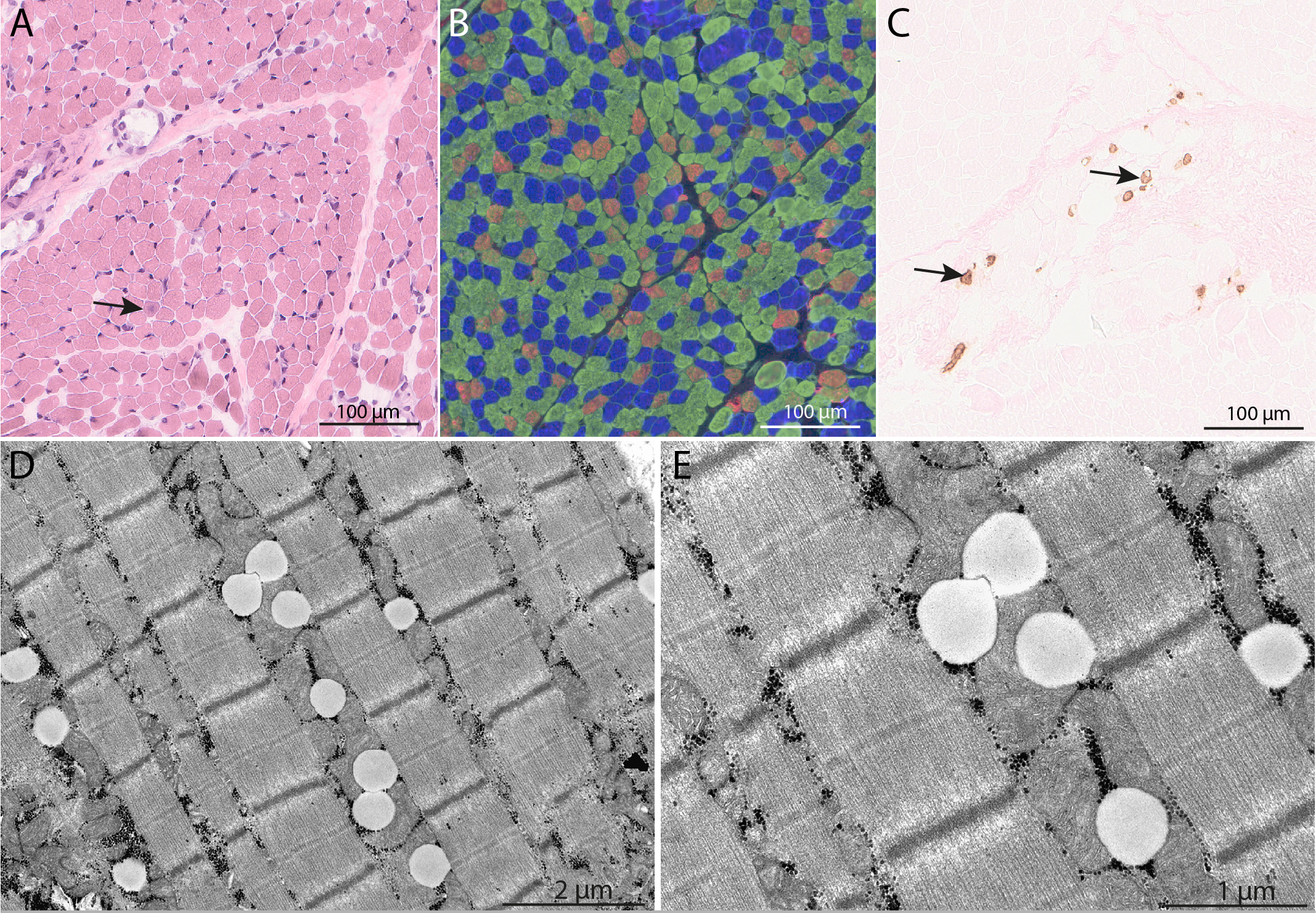


**Supplementary Figure 1. Histopathology in muscle tissue in patient II:8.** (A) Increased variability of fiber size and some basophilic fibers with central nuclei (arrow). (B) Muscle fiber typing showing variation in fiber size among type 1 (blue), type 2A (green) and type 2B (red) fibers. (C) Immunohistochemical staining of embryonic myosin heavy chain showing scattered regenerating muscle fibers (arrows). (D and E) Electron micrographs of a longitudinal section of skeletal muscle showing a region with numerous intermyofibrillar mitochondria and slightly increased amount of fat droplets. The overall structure appears within the normal range.


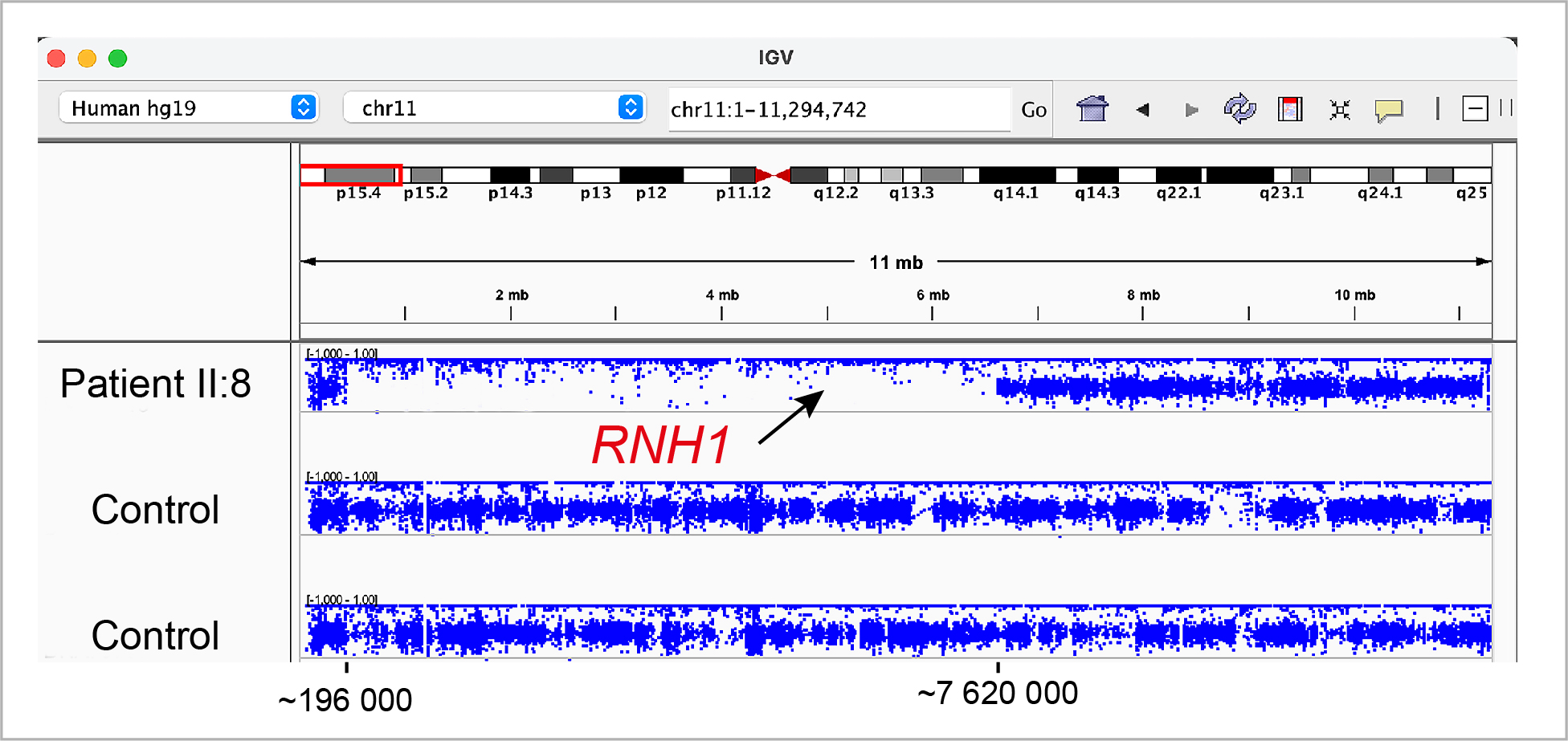


**Supplementary Figure 2.** WGS data displaying a region of 7.2 Mb with loss-of-heterozygosity on chromosome 11 harboring *RNH1* in patient II:8, compared to data from two control subjects.


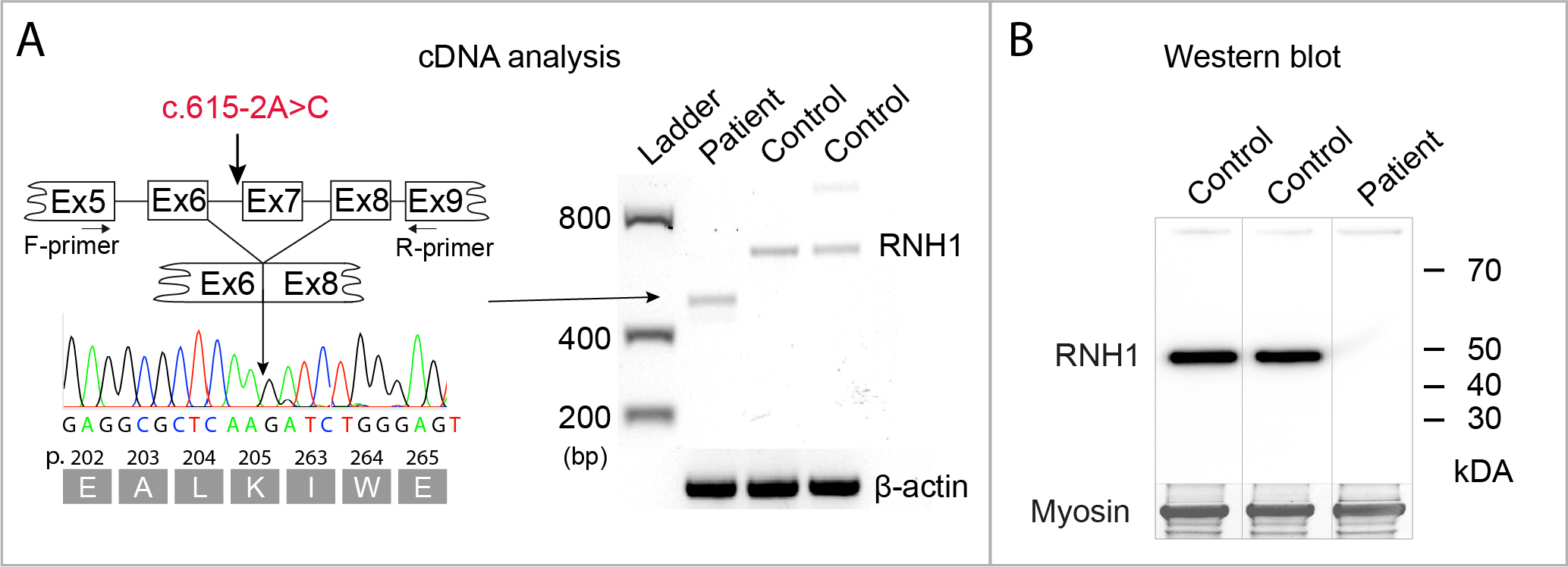


**Supplementary Figure 3. RNH1 analysis in muscle specimens.** (A) Schematic illustration showing the splice-site effect of the c.615-2A>C variant as investigated with reverse transcriptase polymerase chain reaction (RT-PC) followed by PCR and Sanger sequencing using forward primer located in exon 5 and reverse primer located in exon 9. In patient II:8 a band with lower molecular size was identified compared to a control samples using cDNA derived from mRNA extracted from skeletal muscle. Sanger sequencing chromatograms showing transcript lacking exon 7. (B) Western blot analysis on muscle lysates from patient II:8 and two age-matched controls showing that RNH1 is completely absent and that no aberrant spliced protein was detected in the patient compared to the control samples. The band corresponding to myosin heavy chain in the Coomassie stained gel was used as loading control. Unedited version of the Western blot is shown in Supplementary Figure 6.


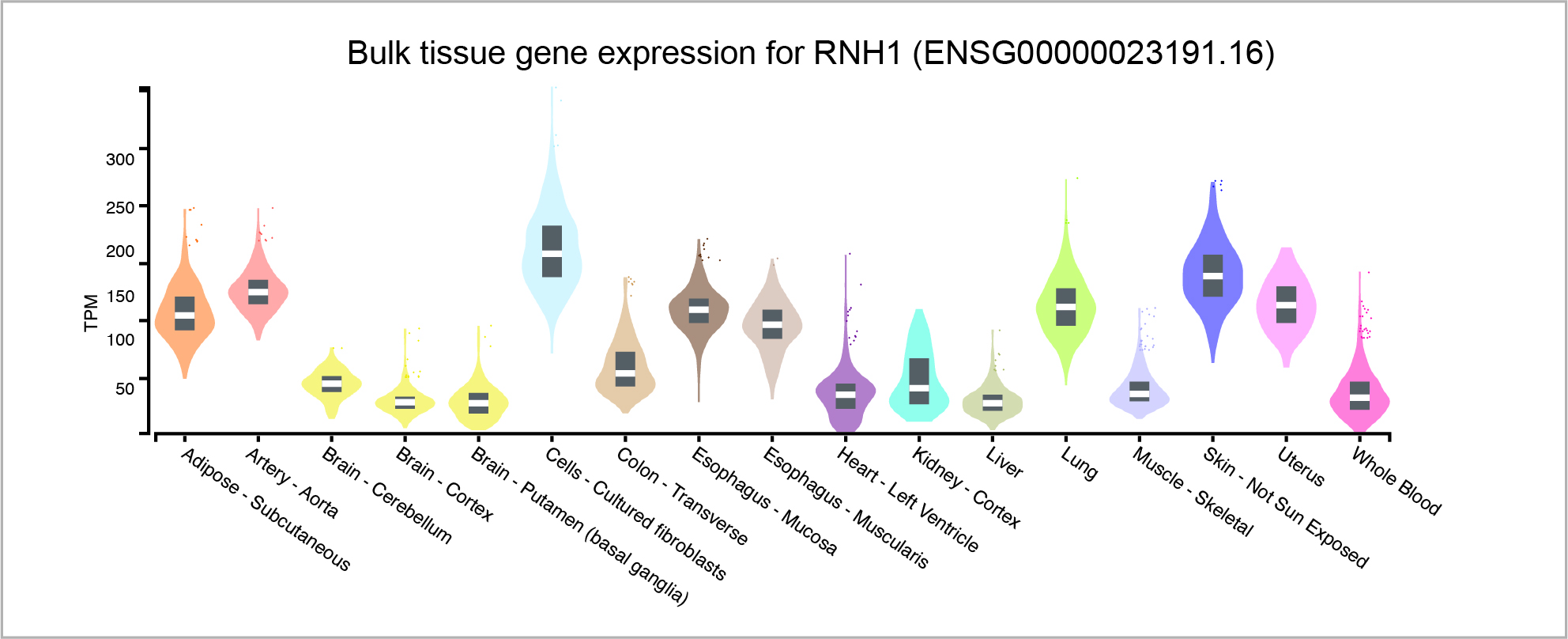


**Supplementary Figure 4.** Gene expression of *RNH1*, obtained from the GTEx Portal; Data Source: GTEx Analysis Release V8 (dbGaP Accession phs000424.v8.p2).


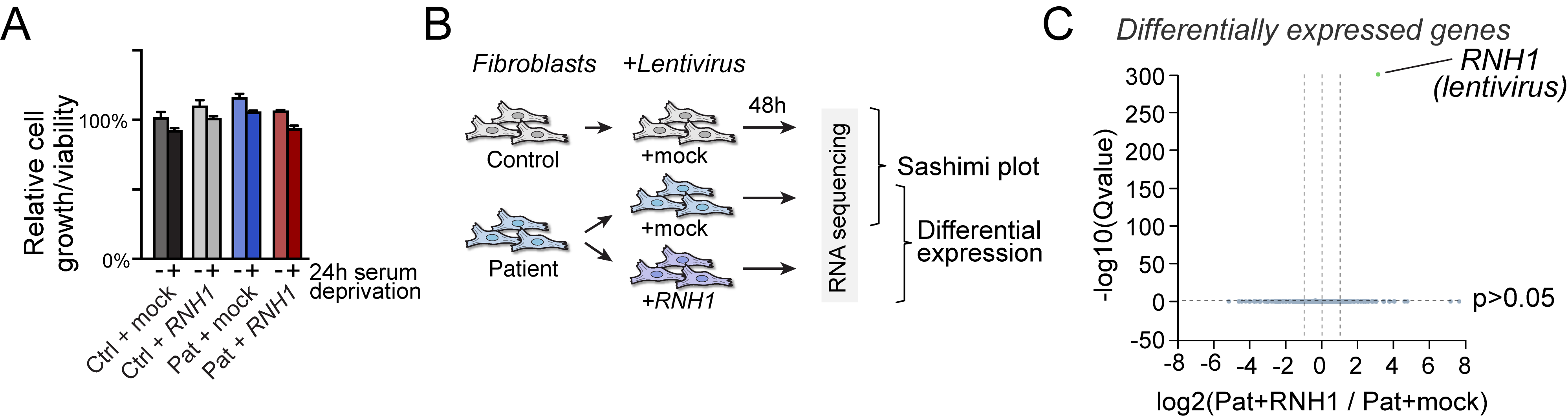


**Supplementary Figure 5. RNA-sequencing in patient-derived fibroblasts.** (A) Analysis of the growth rate/viability of transduced patient-derived fibroblasts under basal and serum-deprived conditions (related to Fig. 4 in the main text). Cells were seeded, grown and analyzed by the MTT assay as outlined in Fig. 4B. Data represented as mean ±standard error of the mean (n = 3). (B) Experimental setup for bulk RNA-sequencing of lentiviral-transduced fibroblasts (n = 2 per condition). (C) Volcano plot of genes differentially expressed between patient cells and patient cells transduced with *RNH1*-expressing lentivirus. The only significant difference was the expected (lentiviral) expression of RNH1 cDNA in complemented cells.


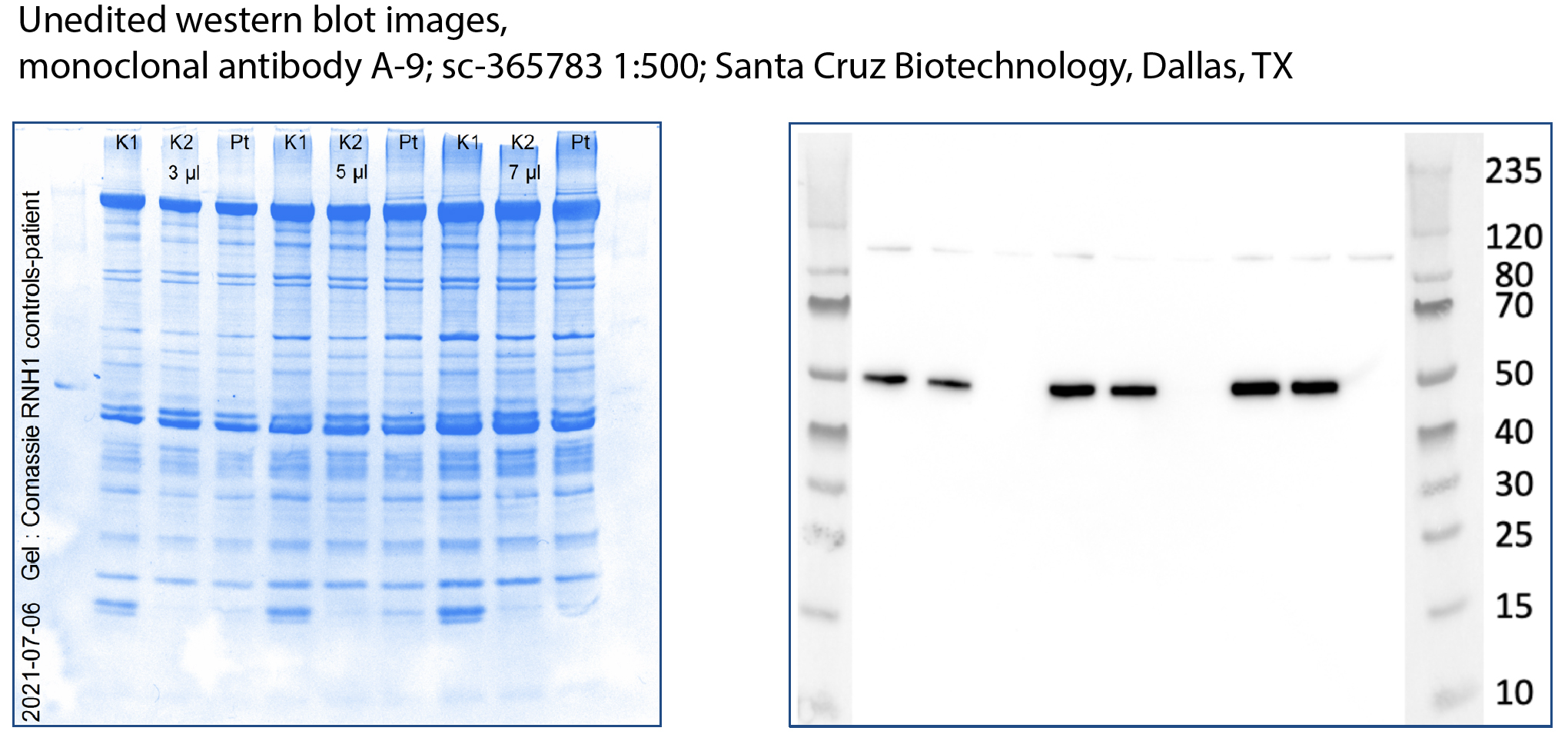


**Supplementary Figure 6. Western blot from muscle specimen in patient II:8.** To the left showing Coomassie stained gel with two aged-matched control (K1 and K2) and patient II:8 (Pt). All three samples are loaded with three different amounts of the protein lysates (3 μl, 5 μl and 7 μl). To the right unedited images corresponding to Supplementary Figure 1G using the monoclonal antibody (A-9; sc-365783 1:500; Santa Cruz Biotechnology, Dallas, TX). Expected size for RNH1 is 50 kDa. Showing that RNH1 is completely absent and that no aberrant spliced protein was detected in patient II:8 compared to two age matched control samples.


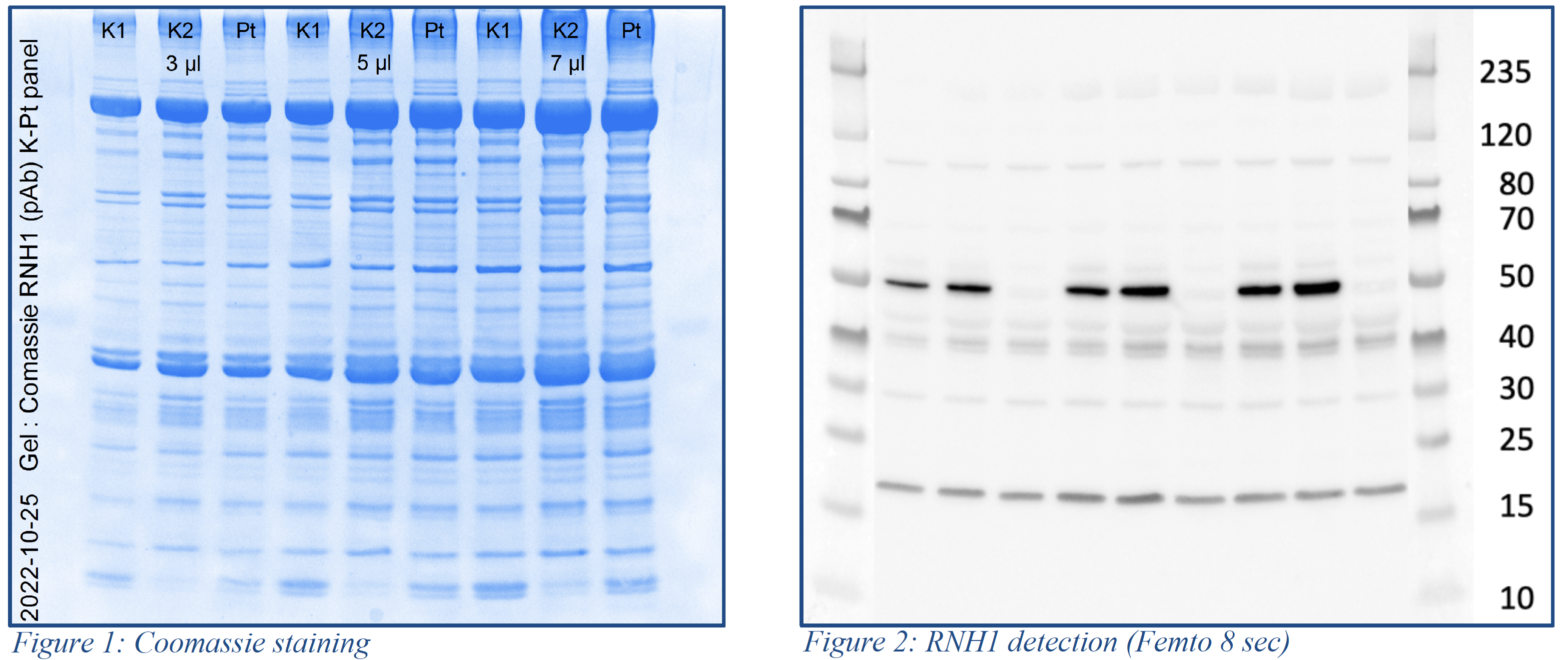


**Supplementary Figure 7. Western blot from muscle specimen in patient II:8.** To the left showing Coomassie stained gel with two aged-matched control (K1 and K2) and patient II:8 (Pt). All three samples are loaded with three different amounts of the protein lysates (3 μl, 5 μl and 7 μl). To the right, unedited images using the polyclonal antibody (anti-RNH1 (B01P, Abnova, H0000050-B01P; pAb); 1:500). Expected size for RNH1 is 50 kDa. Showing that RNH1 is completely absent and that no aberrant spliced protein was detected in patient II:8 compared to two age matched control samples.

**References**

1. Rentzsch P, Schubach M, Shendure J, Kircher M. CADD-Splice-improving genome-wide variant effect prediction using deep learning-derived splice scores. Genome Med. 2021;13(1):31.

2. Adzhubei IA, Schmidt S, Peshkin L, Ramensky VE, Gerasimova A, Bork P, et al. A method and server for predicting damaging missense mutations. Nat Methods. 2010;7(4):248-9.

3. Lee W, Zhang Y, Mukhyala K, Lazarus RA, Zhang Z. Bi-directional SIFT predicts a subset of activating mutations. PLoS One. 2009;4(12):e8311.

4. Phillips JS, Dudík M, E. SR. [Internet] Maxent software for modeling species niches and distributions (Version 341) Available from url: <http://biodiversityinformaticsamnhorg/open_source/maxent/>.

5. Ioannidis NM, Rothstein JH, Pejaver V, Middha S, McDonnell SK, Baheti S, et al. REVEL: An Ensemble Method for Predicting the Pathogenicity of Rare Missense Variants. Am J Hum Genet. 2016;99(4):877-85.

6. Jagadeesh KA, Wenger AM, Berger MJ, Guturu H, Stenson PD, Cooper DN, et al. M-CAP eliminates a majority of variants of uncertain significance in clinical exomes at high sensitivity. Nat Genet. 2016;48(12):1581-6.

7. Li R, Li Y, Kristiansen K, Wang J. SOAP: short oligonucleotide alignment program. Bioinformatics. 2008;24(5):713-4.

8. Langmead B, Salzberg SL. Fast gapped-read alignment with Bowtie 2. Nat Methods. 2012;9(4):357-9.

9. Love MI, Huber W, Anders S. Moderated estimation of fold change and dispersion for RNA-seq data with DESeq2. Genome Biol. 2014;15(12):550.
